# Osteoporotic Goat Spine Implantation Study Using a Synthetic, Resorbable Ca/P/S-Based Bone Substitute

**DOI:** 10.3389/fbioe.2020.00876

**Published:** 2020-08-04

**Authors:** Bing-Chen Yang, Sheng-Min Lan, Chien-Ping Ju, Jiin-Huey Chern Lin

**Affiliations:** ^1^Department of Materials Science and Engineering, College of Engineering, National Cheng-Kung University, Tainan, Taiwan; ^2^Department of Orthopedics, National Cheng-Kung University Hospital Dou-Liou Branch, Yunlin, Taiwan

**Keywords:** Ca/P/S-based, bone substitute, osteoporosis, animal study, histomorphometry

## Abstract

One primary purpose of the present study is to clarify whether the highly porous, resorbable Ca/P/S-based bone substitute used in this study would still induce an osteoporotic bone when implanted into the osteoporotic vertebral defects of ovariectomized (OVX) goats, or the newly-grown bone would expectantly be rather healthy bone. The bone substitute material used for the study is a synthetic, 100% inorganic, highly porous and fast-resorbable Ca/P/S-based material (Ezechbone^®^ Granule CBS-400). The results show that the OVX procedure along with a low calcium diet and breeding away from light can successfully induce osteoporosis in the present female experimental goats. The histological examination reveals a newly-formed trabecular bone network within the surgically-created defect of the CBS-400-implanted (OVX_IP) goat. This new trabecular bone network in the OVX_IP goat appears much denser than the OVX goat and comparable to the healthy control goat. Histomorphometry show that, among all the experimental goats, the OVX_IP goat has the highest trabecular thickness and lowest trabecular bone packet prevalence. The differences in trabecular plate separation, trabecular number and trabecular bone tissue area ratio between the OVX_IP goat and the control goat are not significant, indicating that the trabecular bone architecture of the OVX_IP goat has substantially recovered to the normal level in about 6 months after implantation without signs of osteoporosis-related delay in the bone maturing process. The quick and nicely recovered trabecular architecture parameters observed in the OVX_IP goat indicate that the present Ca/P/S-based bone substitute material has a high potential to treat osteoporotic fractures.

## Introduction

Osteoporosis has become a major public health issue in the world today (Curtis et al., 2017). It was estimated that about half women and one fifth men older than 50 could suffer from one of osteoporosis-related fractures in their lifetime (Sözen et al., 2017). Aging, immobility and menopause are known as the most common causes of osteoporosis. Other reported factors that may increase osteoporosis risks include inadequate nutrition, cigarette smoking, alcohol abuse, hypercortisolism, hyperthyroidism, primary hyperparathyroidism, hypogonadism, hypopituitarism, inherited osteoporosis, excessive exposure to certain drugs such as glucocorticoids (Eastell, 2017). It is known that reduced estrogen at menopause could accelerate bone loss in women, and that estrogen deficiency might be more important than testosterone deficiency in causing bone loss in aging men (Falahati-Nini et al., 2000). Aging can shift mesenchymal stem cell (MSC) differentiation bias, facilitating MSC differentiation into adipocytes instead of osteoblasts (Rosen et al., 2009). The treatment for aging-related osteoporosis has largely been focusing on the stimulation of osteoblast activity and/or the inhibition of osteoclast activity. Both osteoclast activity-suppressing antiresorptive agents and osteoblast activity-enhancing anabolic agents have been developed. The antiresorptive agents include estrogen, selective estrogen receptor modulator (SERM), calcitonin, bisphosphonates and denosumab.

Bone grafting, when properly used, can be very effective in reducing the healing time of musculoskeletal injuries (De Long et al., 2007). The demands of bone grafting increase especially in osteoporosis-related fractures because the process of fracture healing can be significantly impaired in osteoporotic bone (Walsh et al., 1997; Kubo et al., 1999; Meyer et al., 2001; Namkung-Matthai et al., 2001; Lill et al., 2003; McCann et al., 2008). Furthermore, the impaired bone healing in fractures increases the demands of fixation, yet the risk of fixation failure increases in osteoporotic bone due to its porous structure and low strength. Under this consideration, a bone void filler-augmented implant seems to be a good option to treat osteoporotic fractures.

Recognized as a gold standard, autografts have been widely used to repair bone defects. It is known that autograft-induced osteogenesis helps healing in osteoporotic fractures, yet the limited available amount of autologous bone, additional surgical procedures, morbidity and risk of harvesting site fracture have prompted practitioners to seek other bone healing-enhancing materials to replace autografts (De Long et al., 2007), such as allografts, xenografts and synthetic materials.

It is known that one major concern regarding allograft and xenograft treatment is the transmission of diseases. Bacterial infections resulting from the implantation of musculoskeletal allografts have been reported since 1953 (Eastlund, 2006). Hinsenkamp et al. (2012) documented cases of the transmission of human immunodeficiency virus (HIV), hepatitis C virus (HCV), human T-lymphotropic virus (HTLV), unspecified hepatitis, tuberculosis and other bacteria via orthopedic surgeries. Delloye et al. (2007) mentioned that the popular dose of 25 kGy is not virucidal for HIV, whose risk prevention largely relies on the screening procedures and inactivating treatments. Pathological prion was identified in bovine bone marrow (Wells et al., 1999) and serum samples (Trieschmann et al., 2005), while proteins were detected in Bio-Oss^®^ (Schwartz et al., 2000) and tibia samples (Murugan et al., 2003). These findings indicate that bovine-derived grafting materials may carry a risk of prion transmission to human patients.

Use of synthetic materials as bone grafts can, by nature, substantially avoid the above-mentioned risks of disease transmission. Calcium phosphate and calcium sulfate, either in cement or granular form, are two of the most popularly-used synthetic materials as bone substitutes. Both materials have been reported capable of stimulating osteogenesis under normal bone metabolism condition (Decker et al., 1995; Walsh et al., 2003; Ricci et al., 2005). Ideally a resorbable bone implant should have a resorption rate comparable to that of the host bone, yet obtaining an optimal resorption rate has always been a great challenge for a resorbable bone substitute material (Blokhuis, 2014). One reasonable approach to obtain a resorbable bone implant with an optimal resorption rate seems to combine two resorbable biomaterials that have inherently different resorption rates (Hu et al., 2010; Chen et al., 2014; Dewi et al., 2015). In general, the resorption rate of calcium phosphate is often too low to efficiently create a space for new bone ingrowth, while calcium sulfate often resorbs too fast and too quickly create space for ingrowth of soft tissues (Moore et al., 2001). For this reason, a proper combination of calcium phosphate and calcium sulfate, such as that used in the present study, seems to be a logical option for use as a resorbable bone substitute.

The question regarding how osteoporosis can influence the bone grafting-involved bone healing process has not been fully answered. The result is rather hard to predict due to the fact that osteoporosis may alter the resorption rate of the implant in different ways (van Houdt et al., 2018). An animal study of Walsh et al. (1997) indicates that the ovariectomy (OVX)-induced osteoporosis could impair the fracture healing in a rat osteopenia model. From their OVX rat model, Kubo et al. (1999) observed some histologically osteoporotic features accompanied with a decreased BMD at the fracture site at 12 weeks post-surgery in their osteoporosis group. Meyer et al. (2001) observed a significantly impaired fracture healing process accompanied with a significantly decreased three-point bending strength in their OVX rats. The biomechanical data of Namkung-Matthai et al. (2001) from the healing femur of their OVX rats also revealed significant decreases in fracture energy, peak failure load and stiffness as compared with the sham operated group. The study of Lill et al. (2003) also demonstrated a delay of fracture healing in the osteoporotic sheep tibiae. The histological, biomechanical and radiological measures of bone union in the study of McCann et al. (2008) again suggest an OVX-related delayed fracture healing process. Cesnjaj et al. (1991) suggested that the interaction between cells and the graft material might be more crucial than the osteoporotic environment itself of the implant site. The authors took demineralized bone matrix (DBM) from both normal and OVX rats and implanted the DBM samples intramuscularly into both normal and OVX rats. Their results show that the DBM taken from the normal rats resulted in good bone formation in both normal and the OVX rats, while the DBM taken from the OVX rats did not assist the osteogenesis process even in the normal rats. These findings clearly suggest that the OVX-induced osteoporotic changes occur not only in the existing bones but also in the newly-formed bones, and that the regenerated bone in an osteoporotic bone defect could still be osteoporotic.

One primary purpose of the present study is to clarify whether the present highly porous, resorbable Ca/P/S-based bone substitute would still induce an osteoporotic bone when implanted into osteoporotic vertebral defects of OVX goats, or expectantly the newly-grown bone would be rather healthy bone due to the proprietarily-designed chemistry and structure of the product. The methods used for the biological performance evaluation include the histological examination of bone/cell morphology and histomorphometry involving the measurements of trabecular thickness (Tb.Th), trabecular plate separation (Tb.Sp), trabecular bone tissue area fraction (B.Ar/T.Ar), trabecular number (Tb.N), trabecular packet wall thickness (W.WI), trabecular bone packet prevalence (TBPP), Euler characteristic (χ) and trabecular bone pattern factor (TBPf) using reflected and transmitted polarized light microscopy.

## Materials and Methods

### Material Used for the Study

**FIGURE 1:**
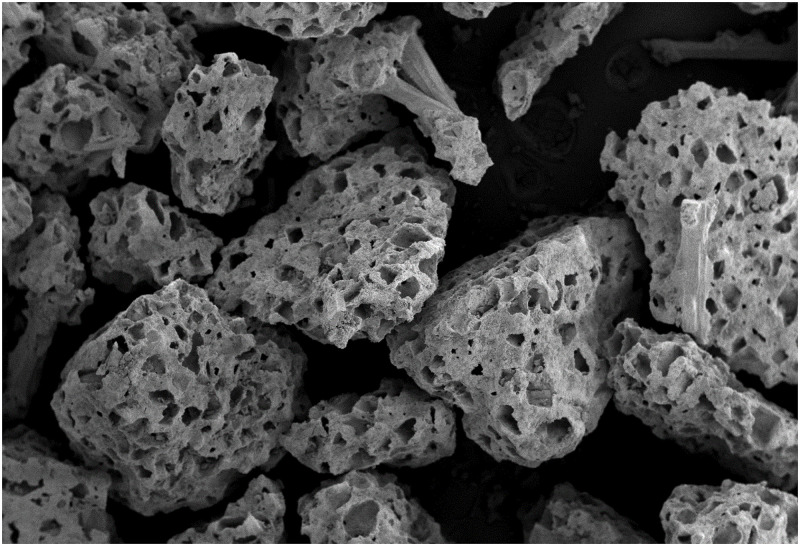
Scanning electron micrograph of the porous granules used for the study.

The material used for the study is a synthetic, 100% inorganic, highly porous (> 70% v/v in porosity), granular (0.4–1.2 mm in diameter) and fast-resorbable Ca/P/S-based bone-substituting material (Ezechbone^®^ Granule CBS-400) developed by a National Cheng-Kung University (NCKU)/Joy Medical Devices Corporation (JMD) joint research project and manufactured at an ISO 13485/GMP-certified facility in Kaohsiung, Taiwan. The highly porous morphology of the granules is shown in Figure 1. The safety and efficacy of the product have been confirmed by a series of chemical/physical characterization and biocompatibility tests such as cytotoxicity, sub-chronic toxicity, intracutaneous reactivity, skin sensitization, genotoxicity and animal implantation.

### Animal Model and Implantation Procedures

The implantation study was conducted according to the procedures approved by the local ethics committee and carried out in accordance with the National Institutes of Health Guide for the Care and Use of Laboratory Animals. Four 2–3 year-old female goats were used for the present study. Except one control goat which was fed and bred normally, the other three goats underwent OVX, fed with a low calcium diet and bred away from light to help induce osteoporosis (Clements et al., 1987; Kubo et al., 1999; Namkung-Matthai et al., 2001). 18 months after the OVX procedure, artificial bone voids in vertebral bodies were surgically created in two OVX-induced osteoporotic goats, while the third goat was sacrificed and designated “OVX goat.” The surgeries were conducted under general anesthesia with local anesthesia. Zoletil 50 (0.5 mL/kg, Virbac, Carros, France) was used as the general anesthesia, while xylocaine (AstraZeneca, Cambridge, England, United Kingdom) was used as the local anesthesia. Implant sites were shaved and cleansed with 70% v/v ethanol and Betadine^®^ (povidone iodine 10% w/v). A midline longitudinal incision was made in the dorsal skin over lumbar vertebra between L1 and L3, wherein left paraspinal muscles were elevated to expose the spinous process, lamina, medial part of the transverse processes and the facet joints, against which a Taylor retractor was placed to maintain the exposure.

**FIGURE 2:**
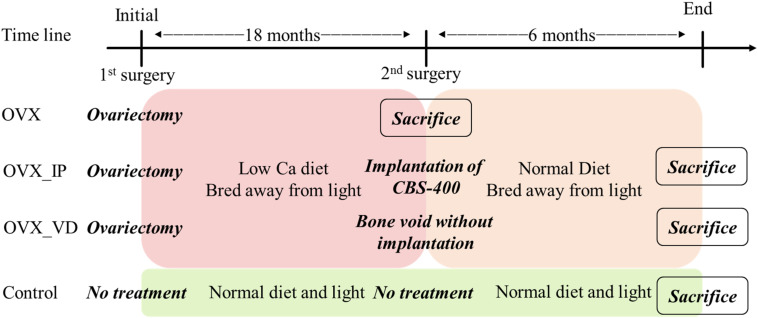
Goat implantation design and surgery timeline.

The cortical bone of the entry site at the junction of transverse process and the base of the superior articular process was opened using a burr. A trans-pedicular tunnel all the way to the vertebral body was subsequently enlarged to an elliptical tunnel with a major axis of 10 mm and minor axis of 8 mm using curettes to avoid breaching the neural canal. To more meaningfully test the healing potential of the implanted porous granules, this tunnel size was larger than the critical size suggested in the literature (5 mm in diameter) (Li et al., 2010b). The created bone void in the vertebral body of one goat was filled with 1.0 mL CBS-400 granules (designated “OVX_IP goat”), while the void of the other goat was left without implantation (designated “OVX_VD goat”). A bone curette of 5 × 8 mm in inner diameter was used to deliver the CBS-400 granules into the surgically-created bone cavities. To improve the handleability of CBS-400, prior to delivery, the loose granules were mixed with saline to form a sticky cluster. During the implantation period all OVX goats were fed a normal calcium diet. The OVX goats were sacrificed at 24 weeks post-operation. A timeline of the treatment and the diet of the experimental goats are shown in Figure 2.

### Histological Examination

**FIGURE 3:**
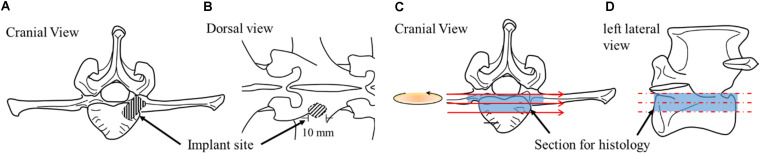
Schematic drawings illustrating implantation site from different perspectives **(A,B)** and sample sectioning for histology **(C,D)**.

After the animals were sacrificed, the lumbar vertebrae were excised immediately and the excess tissues were removed. In the control goat and the OVX goat without surgical treatment, L3 vertebral bodies were taken for the study. The vertebral bodies were taken from the sections close to the middle of the implant site in the vertebral body, as illustrated in Figure 3. The samples were sectioned using a low-speed diamond blade (IsoMet Blade, Buehler, Illinois, United States).

The sectioned samples were fixed in 10% w/v neutral buffered formalin (NBF) (pH 7.0) for 3 days, dehydrated in increasing grades of ethanol, and embedded in Buehler EpoxiCure 2 resin. Each embedded sample was sectioned into two portions, therein one portion was used to prepare thin sections for polarized transmitted light microscopy and the other portion was used for reflected light microscopy. The transmitted polarized light microscopy was applied to examine bone ingrowth, bone/implant interface, cell type and collagen fiber orientations in bone. The samples were ground using silicon carbide grit paper and polished by wet-cloth sequentially with 1.0, 0.3, and 0.05 μm Al_2_O_3_ powder. The polished surfaces were glued to slides with resin. After the resin was hardened, the samples were thinned to a final thickness of about 100 μm, followed by toluidine blue (TB) staining and sealing with Permount (Fisher Scientific, Fair Lawn, NJ, United States). A polarized light microscope (DM2500P, Leica Co., Germany) was used for the study. TB has been recognized as one of the few dyes that can penetrate the epoxy resin to stain the tissue section and commonly used in staining undecalcified bone sections (Lowe et al., 2018). The relatively mature lamellar bones are aligned in layers, wherein collagen bundles are parallel to each other and generate a birefringence effect under the polarized light, allowing easy identification of their existence (Bullough, 2010; Warshaw et al., 2017). To more clearly examine the orientations of the collagen fibers in trabeculae and distinguish between implanted granules and the new bone, 100 μm thin sections were commonly used for transmitted polarized light microscopy due to the difficulty in detecting collagen fiber orientations using the reflection mode. Nevertheless, the transmitted polarized light microscopy used in the present study can easily distinguish lamellar bone from woven bone due to their different birefringent effects (Gunson et al., 2015; Ip et al., 2016).

To study histomorphometry and determine the architectural parameters of the trabeculae, on the other hand, a reflected light was applied for its clearer implant/bone interface appearance than the transmitted light partly due to the elimination of thickness interference. The sections for the reflected light examination were prepared by the same grinding and polishing procedures. To enhance resolution and at the same time to obtain an overall picture of the trabecular features along with the implant resorption behavior, 100 + micrographs were taken sequentially on each section being examined. These 100 + micrographs were then superimposed to form a large composite picture covering the entire implant/bone cross-section using Leica Application Suite software.

To compare the morphological information between optical microscopy and SEM backscatter election microscopy, an SU3500 SEM (Hitachi, Tokyo, Japan) operated at 10 kV on a backscatter mode was used. The samples for the SEM examination were obtained from the OVX_IP goat. The samples for the SEM were prepared following the same procedures as that for the reflected light microscopy, except that the samples for SEM were further sputter-coated with a thin layer of gold on the surface to avoid electric charging.

### Histomorphometry

The architectural parameters of the trabeculae were evaluated using an image analysis system (ImageJ, National Institute of Mental Health, United States). These architectural parameters include trabecular thickness (Tb.Th), trabecular plate separation (Tb.Sp), trabecular number (Tb.N), trabecular bone tissue area fraction (B.Ar/T.Ar), Euler characteristic (χ) and trabecular bone pattern factor (TBPf). For the reflected light examination and measurements of the wall thickness (W.WI) and trabecular bone packet prevalence (TBPP) (number of trabecular packets per bone area), the 0.05 μm Al_2_O_3_-polished samples were etched by 1% acetic acid for 2 min to help reveal such feature as cement lines.

For the measurement of W.WI, only complete trabecular bone packets at trabecular surfaces or complete osteons without resorption lacunae or osteoid seam were measured. The distance between the cement line and the border facing the marrow was measured on 4 equidistant places at each packet and their average was taken as the W.WI of the packet. At least 90 packets were measured on each sample and their average was regarded as the W.WI of that sample (Lips et al., 1978; Darby and Meunier, 1981). For the measurement of TBPP, all packets including superficial packets with any portions extending to the bone surface and deep packets completely encapsulated by cement lines in the interior of the trabeculae were counted and divided by the measured bone area (Skedros et al., 2012). The TBPfs and Euler characteristics were measured according to the methods described by Hahn et al. (1992) and Boutry et al. (2003), respectively. The ratio of the Euler characteristics to the area of the interested region (χ/ROI, mm^–2^) was used to normalize the influence of the area observed (Boutry et al., 2003). According to this method, a structure with higher connectivity would have lower values of χ/ROI and TBPf.

### Statistical Analysis

The quantitative data are presented as means ± standard deviations. In each animal, four sections were examined for bone histomorphometry except W.WI and TBPP, which were measured from one section. For TBPP, six sites were examined in each section. Unpaired Student’s *t*-test was used to compare the differences in histomorphometry between the experiment groups and the control group, wherein significance was considered at *p* < 0.05.

## Results

### Histology and Bone Morphology

**FIGURE 4:**
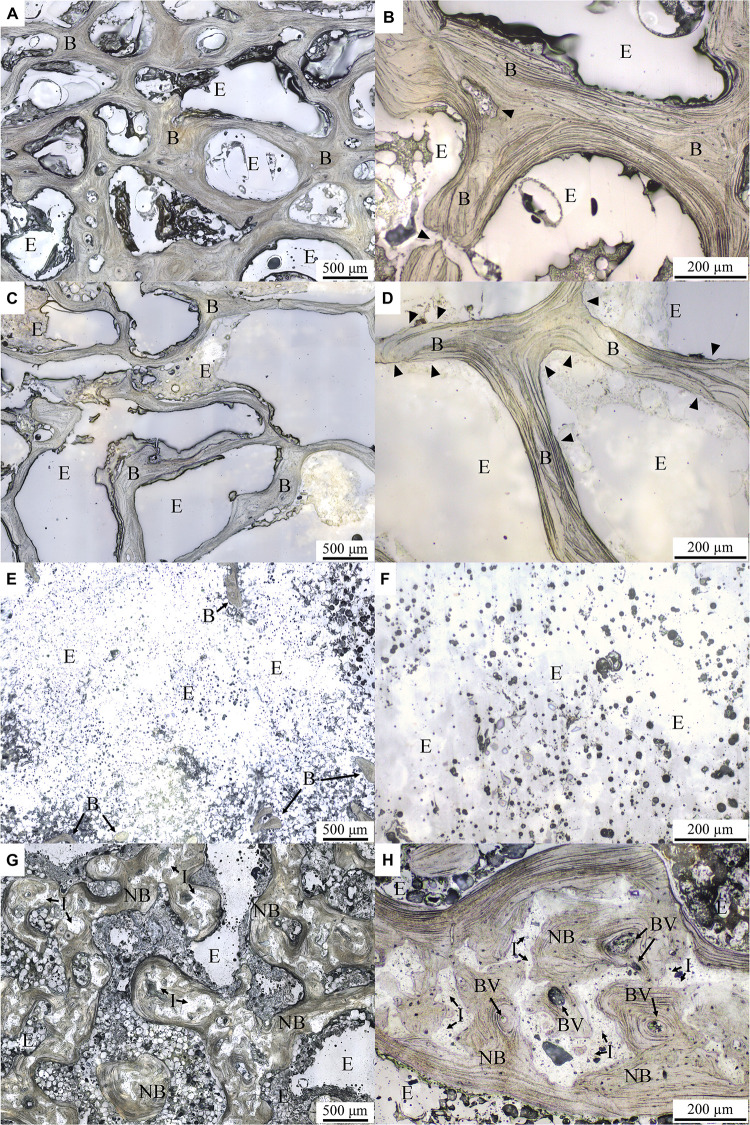
Histological micrographs under normal reflected light with different magnifications. **(A,B)** control goat; **(C,D)** OVX goat; **(E,F)** OVX_VD goat; **(G,H)** OVX_IP goat. I, residual implant; NB, new bone; B, bone; E, epoxy; BV, blood vessel. Black arrowheads indicate the surface of the bone resorbed by osteoclast leaving behind an etching mark.

Typical lower-magnification histological micrographs under normal reflected light of the control, OVX, OVX_VD, and OVX_IP goats are shown in Figures 4A,C,E,G, respectively. As clearly demonstrated in the micrographs, the trabecular structure in the OVX goat is much more porous and the trabeculae therein are much thinner than the control goat. The newly-developed trabecular bone network in the OVX_IP goat is much denser than the OVX goat and comparable to the healthy control goat. The large empty space observed in the OVX_VD goat indicates that the size of the surgically-created defect is large enough so that it cannot be repaired by the normal bone healing mechanisms. Typical higher-magnification histological micrographs under normal reflected light of the control, OVX, OVX_VD, and OVX_IP goats are given in Figures 4B,D,F,H, respectively. It is interesting to see that, in the OVX_IP goat, a new bone network is observed to have developed within the defect, wherein the barely identifiable tiny, numerous residual implanted granules are intimately blended in the surrounding new bone throughout the newly-established lamellar bone structure. Seen throughout the entire implantation site is tight, continuous interface between the implant and the host bone without interposition of fibrous tissues. Resorption by osteoclast, which is evidenced by the etched features on bone surface, is easily observed in the OVX goat, but hardly found in the control and OVX_IP goats. Few blood vessels are observed in the trabeculae of the control and OVX goats. In the trabecular bone structure of the OVX_IP goat, on the other hand, numerous blood vessels and newly-formed trabecular packets surrounding implanted granules are observed. Black arrow head indicated the bone surface that was resorbed by osteoclast, leaved a sign of etching.

**FIGURE 5:**
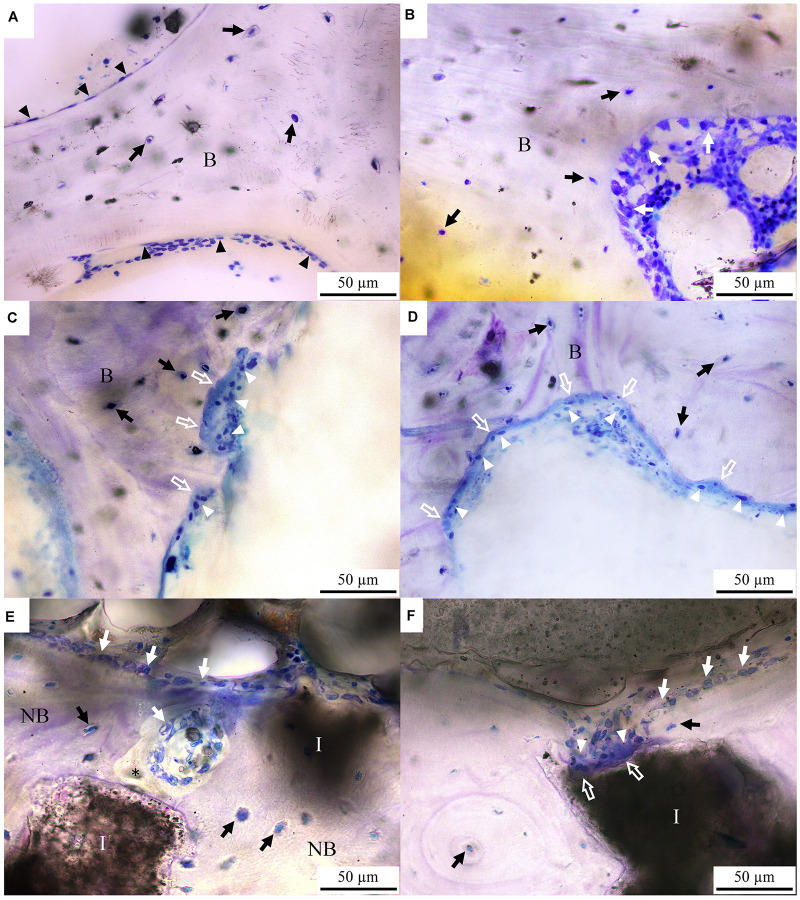
Toluidine blue-stained histological micrographs under normal transmitted light. **(A,B)** control goat; **(C,D)** OVX goat; **(E,F)** OVX_IP goat. B, bone; NB, new bone; I, residual implant; asterisk, osteoid; white arrow, osteoblast; black arrow, osteocyte; white arrow head, osteoclast; black arrow head, lining cell; white hollow arrow, Howship lacunae.

The bone cell morphologies of the control, OVX and OVX_IP goats examined by normal transmitted light are presented in Figure 5. In the control goat, lining cells are observed to cover most bone surfaces (Figure 5A). The formation of new bone by osteoblasts are observed in the control goat (Figure 5B). In the OVX goat, however, extensive bone resorption featured by the Howship lacunae and clusters of osteoclasts on the etched bone surface is observed substantially without signs of new bone formation (Figures 5C,D). The different levels in transparency between the bone tissue and the implanted granules easily reveal the implant residues that are intimately embedded in the surrounding bone tissues. Many mature osteocytes are also observed beside the residual granule particles. It is interesting to note that, in the OVX_IP goat, the newly-formed bone adjacent to the implant is dense, extensive and with numerous osteocytes present in the bone matrix (Figures 5E,F). Patterns of resorption by osteoclasts like the Howship lacunae are observed at some edges of residual granules. The resorption of granules by osteoclasts and new bone formation by osteoblasts are ongoing in the implantation region (Figure 5F).

**FIGURE 6:**
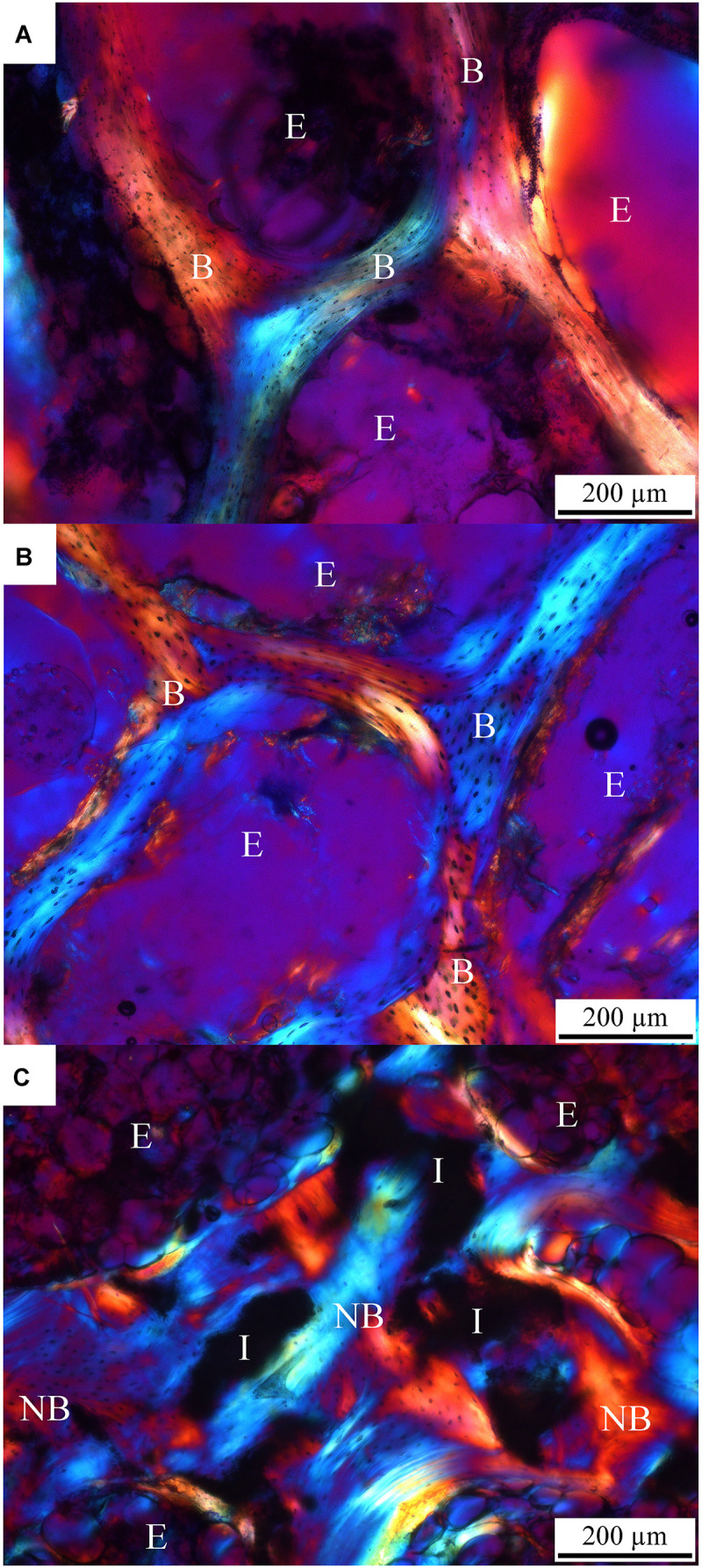
TB-stained histological micrographs under polarized transmitted light. **(A)** control goat; **(B)** OVX goat; **(C)** OVX_IP goat. I, residual implant; NB, new bone; B, bone; E, epoxy; blue color, bone with oriented collagen; orange color, bone with perpendicularly oriented collagen; purple color, randomly oriented resin.

Typical transmitted polarized light micrographs of the control, OVX and OVX_IP goats are shown in Figure 6. Under this polarized light, the blue and orange regions represent two perpendicular fiber orientations, while the purple color represents a rather randomly-orientated region without specific fiber features. Despite their much different porosity levels, the control (Figure 6A) and OVX goats (Figure 6B) show similar trabecular orientation features under the polarized light. It is interesting to see that, compared to the control and OVX goats, a more randomly-oriented, smaller-sized and apparently more active lamellar bone morphology is observed in the OVX_IP goat (Figure 6C). These lamellar bones are often observed to be parallel to the implant surface. The dark gray-appearing implant residues are observed to be nicely embedded in the surrounding intertwining bone, forming a dense bone/implant composite trabecular bone network. Woven bone is substantially absent in the OVX_IP goat. Figure 6 further shows that the proportion of the new bone (represented by the blue and red bands) in the OVX_IP goat is much larger than those in the OVX and control goats, indicating a highly osteoconductive characteristic of the present CBS-400 implant.

### Histomorphometry and Changes in Trabecular Architecture Parameters

**FIGURE 7:**
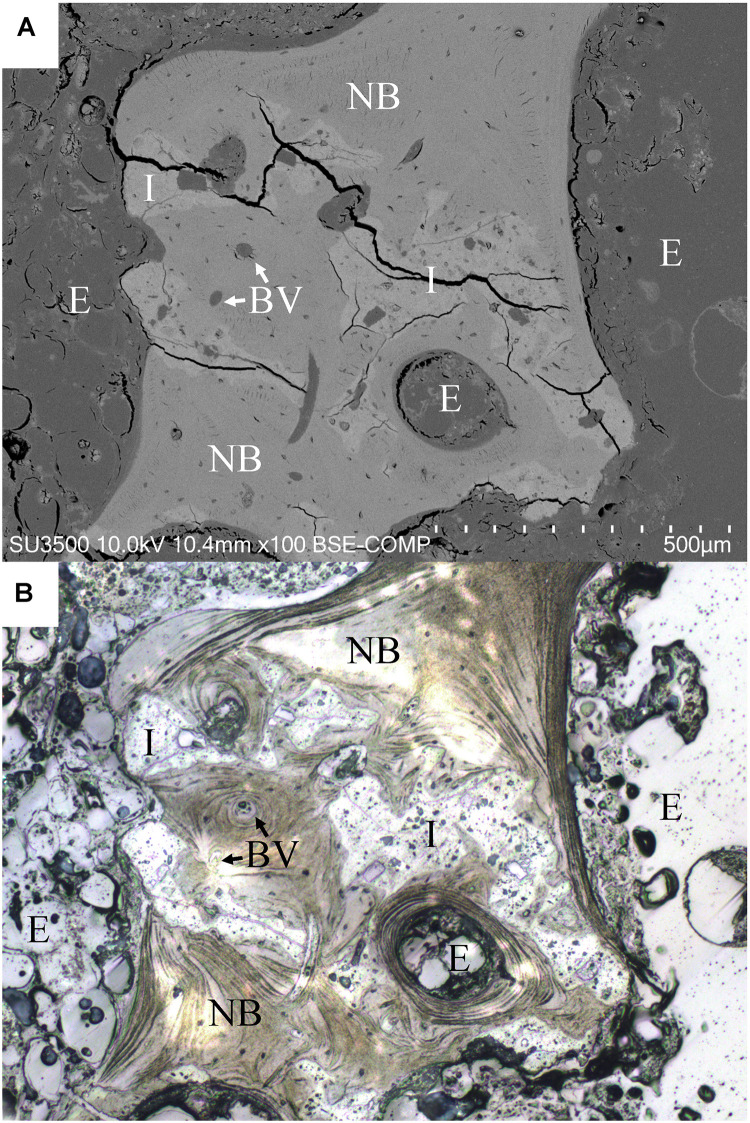
Comparison of backscatter election image (BSI) in scanning electron microscope **(A)** and optical microscopy of the same area **(B)**. Both BSI and optical images can clearly reveal bone and residual implant, yet the optical image is better in revealing bone features like laminar structure. I, residual implant; NB, new bone; E, epoxy; BV, blood vessel.

As mentioned in section “Materials and Methods,” SEM backscatter election microscopy was performed to compare its morphological information with that of the optical microscopy used in the study. Figure 7 demonstrates the morphologies obtained from both techniques of the same area. The figure indicates that both optical image and backscatter election image (BSI) can clearly reveal the bone and residual implant, yet the optical image is consistently better in revealing certain crucial bone features, such as laminar structure. For the sake of obtaining quantitative information, the optical microscopy was used for all the measurements in this study.

**FIGURE 8:**
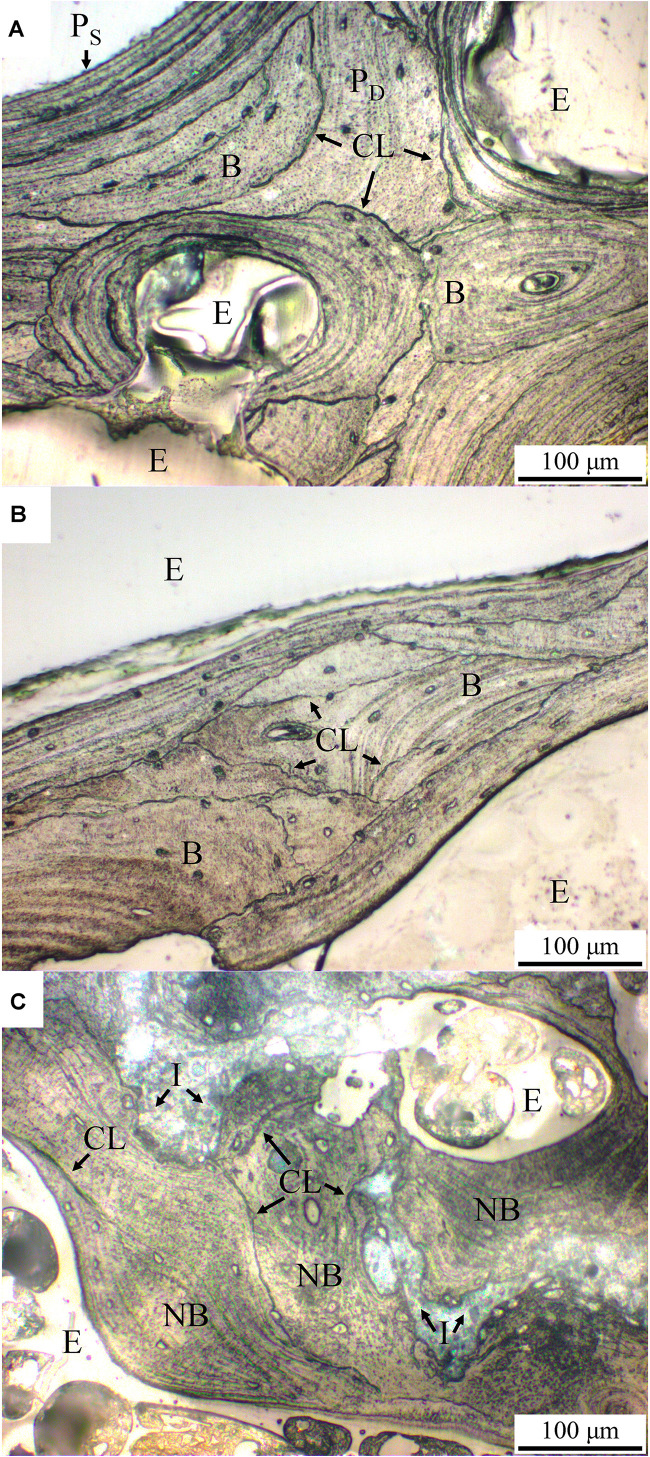
Histological micrographs of acetic acid-etched samples under normal reflected light. **(A)** control goat; **(B)** OVX goat; **(C)** OVX_IP goat. P_D_, deep trabecular packet; P_S_, superficial trabecular packet; CL, cement line; I, residual implant; NB, new bone; B, bone; E, epoxy.

As can be seen in Figure 8, the trabecular bone structure in both control and the OVX goats show numerous short, deep trabecular packets surrounded by the cement lines in the inner layer of the trabecular bone as well as long superficial trabecular packets in the outer trabecular bone, as respectively indicated by P_D_ and P_S_ in Figure 8A. The patterns of trabecular packets in the control and OVX goats are similar (Figures 8A,B). On the other hand, in the trabecular bone structure of the OVX_IP goat, numerous newly-formed trabecular packets with relatively thick walls surrounding the implanted granules are observed. Compared to the control and OVX goats, the number of deep and superficial trabecular packets in the OVX_IP goat is smaller, and so is the number of cement lines (Figure 8C).

**FIGURE 9:**
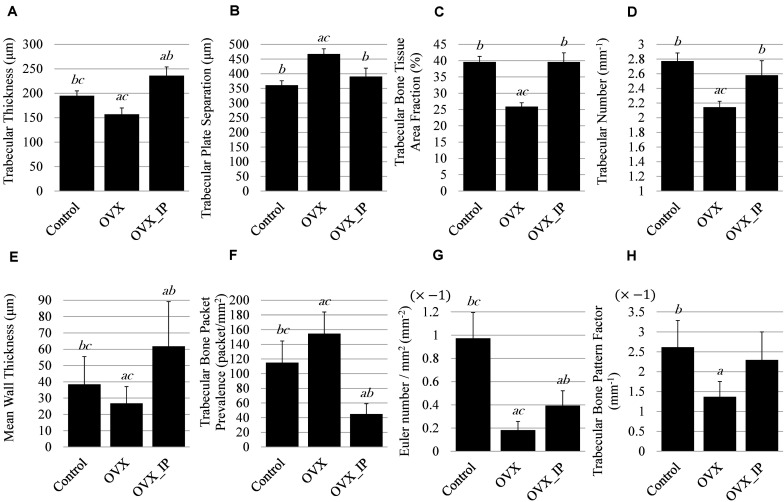
Histomorphometry parameters of control, OVX_IP and OVX goats. **(A)** trabecular thickness; **(B)** trabecular plate separation; **(C)** trabecular bone tissue area fraction; **(D)** trabecular number; **(E)** wall thickness; **(F)** trabecular bone packet prevalence; **(G)** Euler number; **(H)** trabecular bone pattern factor. Symbols *a, b*, and *c* indicate that the mean value has a significant difference (*p* < 0.05) compared to control, OVX and OVX_IP goats, respectively.

Figure 9 summaries the results of histomorphometry and the changes in trabecular architecture parameters of the control, OVX and OVX_IP goats. As indicated in Figure 9A, the OVX_IP goat has the highest average trabecular thickness (236 μm), while the OVX goat has the lowest trabecular thickness (157 μm). The trabecular thickness of the control goat is in between (195 μm). Figure 9B indicates that the average trabecular plate separation of the OVX goat (467 μm) is much larger than those of the control goat (361 μm) and the OVX_IP goat (390 μm). The average trabecular bone tissue area ratio and trabecular number of the OVX goat (25.9% and 2.1 mm^–1^, respectively) are significantly lower than those of the control goat (39.6% and 2.8 mm^–1^, respectively) and the OVX_IP goat (39.6% and 2.6 mm^–1^, respectively) (Figures 9C,D). Compared to the control goat, the OVX goat shows significant difference in trabecular thickness (by 19.8% reduction), trabecular number (by 22.7% reduction), trabecular bone tissue area ratio (by 34.6% reduction) and trabecular plate separation (by 29.3% increase). On the contrary, the differences in trabecular plate separation, trabecular number and trabecular bone tissue area ratio between the control and OVX_IP goats are not significant. The trabecular thickness in OVX_IP goat is significantly larger (by 21.0%) than that of the control.

Among the three experimental goats, the OVX goat has the lowest average mean wall thickness (26.8 μm) and the highest trabecular bone packet prevalence (154.6 packets/mm^2^), while the OVX_IP goat has the highest wall thickness and lowest trabecular bone packet prevalence (61.8 μm and 44.9 packets/mm^2^, respectively). The control goat has an average mean wall thickness (38.4 μm) and trabecular bone packet prevalence (114.9 packets/mm^2^) in between the OVX and OVX_IP goats (Figures 9E,F). Figures 9G,H show that the average Euler number and trabecular bone pattern factor of the OVX goat (−0.182 mm^–2^ and −1.369 mm^–1^, respectively) are also much higher than the control goat (−0.973 mm^–2^ and −2.616 mm^–1^, respectively) and the OVX_IP goat (−0.394 mm^–2^ and −2.295 mm^–1^, respectively). The trabecular bone pattern factors of the control and OVX_IP goats are not significantly different. The connectivity of trabeculae in the OVX goat is significantly lower than the control goat, as indicated in its higher Euler number and TBPf value. On the contrary, the new bone connectivity of the present OVX_IP goat is significantly higher than that of the OVX goat.

## Discussion

Different animal models have been used to study osteoporosis, including rodents and larger animals such as dogs, pigs, sheep, goats, and primates (Dion et al., 2011). Although the well-established OVX rat model, which can mimic postmenopausal bone change (Dion et al., 2011), has been used for the study of cancellous bone changes in humans, the bone metabolism of rats is very different from humans (Wronski et al., 1989). Other problems with the OVX rat model include the lack of Haversian or intracortical remodeling, and the absence of decreased bone formation during the late stages of estrogen deficiency (Jee and Yao, 2001). Apparently, use of larger species, whose bone goes through a coordinated process of bone resorption and new bone formation like humans, would be a better choice for the animal study of osteoporosis. Due to the similarity in biochemical and histopathologic features between osteoporotic goats and humans (Leung et al., 2001), the OVX-induced osteoporotic goat model has been established to assess the healing potential of different bone grafting materials (Leung et al., 2001, 2006; Li et al., 2010b; Cao et al., 2012; Yu et al., 2015).

According to the established protocols, i.e., an OVX procedure along with a low calcium diet and being bred away from light, osteoporosis is successfully induced in the female goats in the present study. The fact that the trabecular structure in the OVX goat is much more porous and its trabeculae are much thinner than the control goat confirm the effectiveness of the present treatment in inducing osteoporosis in the experimental female goats. Furthermore, the absence of trabecular bone within OVX_VD goat indicates that the surgically-created defect is a critical size defect.

The observed clusters of osteoclasts on the etched bone surface in the OVX goat was also reported by Kubo et al. (1999), which might be a result of a deficiency of estrogen restraining the activity of osteoclasts (Demontiero et al., 2012). On the other hand, the coupling of bone resorption and formation suggests that residual granules are possibly resorbed by osteoclasts at the same time when osteoblasts produce new osteoids.

Normally, the human skeleton is composed of about 80% cortical bone and 20% trabecular bone, yet osteoporotic fractures tend to occur at sites comprising more trabecular bone than cortical bone. The osteoporosis-involved structural changes in trabeculae, such as trabecular plate thinning and loss of bone volume/mass, can reduce bone strength, which, in turn, can cause fractures in the osteoporotic bone (Eastell, 2017). Histomorphometry, which is accepted for the study of bone metabolism in animal studies (Dion et al., 2011), is used in the present experimental goats to assess the changes in architecture parameters.

As demonstrated in Figure 9, compared to the control goat, the OVX goat shows significant reductions in Tb.Th, Tb.N, and B.Ar/T.Ar accompanied with an increase in Tb.Sp. Similar changes were observed by Yu et al. (2015), wherein a 11.3% decrease in Tb.Th, 36.7% decrease in Tb.N and 37.1% decrease in B.Ar/T.Ar accompanied with a 62% increase in Tb.Sp in the trabeculae of lumbar vertebral body of 7 OVX goats were observed at 24 months post-ovariectomy. No significant differences in Tb.N, Tb.Sp or B.Ar/T.Ar between the OVX_IP goat and the control goat indicate that the architecture of trabecular bone of the CBS-400-implanted OVX_IP goat has substantially recovered to normal levels.

Trabecular connectivity is also an important parameter in trabecular architecture. The connectivity of trabeculae in the OVX goat is significantly lower than the control goat. This impairment is commonly observed in estrogen deficiency-induced osteoporosis (Yu et al., 2015). The new bone connectivity of the present OVX_IP goat is significantly higher than that of the OVX goat, indicating an improvement in bone connectivity with CBS-400 implant. The somewhat larger Tb.Th observed in the OVX_IP goat than the control goat is believed to be a result of the presence of numerous tiny implant residues which are not yet completely resorbed, as clearly revealed in Figure 4H.

Depending on the fracture type and treatment, different calcium-based bone substitute-involved bone healing mechanisms have been proposed in the literature. Some *in vitro* and *in vivo* studies indicated that calcium phosphate might induce osteogenic differentiation of MSCs leading to new bone formation in bone defects (Müller et al., 2008; Song et al., 2013). In an *in vitro* study, Chen et al. (2017) found that the bone marrow-derived MSCs could be attracted to calcium phosphate to promote MSC differentiation toward vascular endothelial cells and help the revascularization process for bone fracture healing. Implanting β-TCP in sheep femur, Decker et al. (1995) observed that osteoblasts with an endothelial cell marker were located adjacent to the endothelial cells in blood vessels at the new bone formation site, thus suggesting that osteogenic cells could be derived from endothelial cells. From these findings it seems reasonable to assume that, at the early stage of bone healing after implantation of a calcium phosphate material, the MSCs recruited from bone marrow or blood migrate to the implant and differentiate into osteoblasts or vascular endothelial cells. Although the exact mechanism of calcium phosphate-promoted osteogenic differentiation of MSCs is not fully established, the relationship between MSCs and the implant-induced elevated extracellular calcium ion concentration may play a major role (Chang et al., 2000).

The bone remodeling process usually takes a relatively long period of time for lamellar bone to gradually replace a calcium phosphate implant and woven bone (Barrère et al., 2006). Woven bones comprising irregularly and loosely-arranged collagen fibers are a form of mineralized tissue commonly seen during the development of the skeleton or in the fracture callus. Lamellar bones, on the other hand, comprise well-aligned collagen fibers which can increase bone strength. The new bone observed in the present OVX_IP goat is primarily lamellar bone aligned parallel to the surface of implanted granules. Woven bone is substantially absent in the OVX_IP goat probably due to the fast bone healing process facilitated by the present Ca/P/S-based implant. Compared to the control and OVX goats, a thicker and more randomly-oriented lamellar bone morphology is seen in the OVX_IP goat due to the reality that the orientations of implanted granules are random in nature. The randomly-oriented implanted granules, in turn, cause the newly-formed lamellar bone, which forms substantially along the implant surface, to be randomly-oriented, too.

Calcium sulfate, a minor component of the present CBS-400 implant, was also reported to be able to enhance the migration of MSCs and recruit osteoprogenitor cells (Aquino-Martínez et al., 2017). Compared to calcium phosphate, the research into calcium sulfate’s effect on bone healing is relatively limited. Ricci et al. (2005) found that, when calcium sulfate was dissolved in body fluid, the released calcium ions could form a calcium phosphate layer bonded to the adjacent new bone. In an *in vitro* study, Carinci et al. (2004) suggested that the precipitation of calcium phosphate on calcium sulfate surface could form an osteoblast-friendly environment, wherein the activities of cell cycle regulation, signal transduction, immunity and production of lysosomal enzymes of osteoblast-like cells could be enhanced. Walsh et al. (2003) hypothesized that the mechanism of calcium sulfate-assisted bone regeneration process might be related to the local acidic environment and the demineralization of adjacent bone to release matrix-bound bone morphogenetic proteins (BMPs) during its dissolution.

Due to the experimental design, the phenomena of recruitment of MSCs and differentiation of osteoblasts at the very early stage of bone healing cannot be examined in the present study. However, the present histological study provides some information related to bone remodeling at the later stages of the bone healing process, wherein osteoclasts absorbing implanted granules and osteoblasts forming new osteoids (Barrère et al., 2006) are demonstrated. The observed wall thickness of the trabecular bone also stores some information about bone formation (Lips et al., 1978). According to Arvidson et al. (2011), the measurement of the wall thickness, which is related to the number and activity of recruited osteoblasts at the early stage of bone formation, would be useful in the evaluation of osteogenic differentiation and osteoblast activity. A decrease in wall thickness is considered a consequence of the natural aging of humans as well as a sign of impaired osteoblast function (Lips et al., 1978; Darby and Meunier, 1981). Similar functional impairment of osteoblasts was also found in osteoporotic patients (Darby and Meunier, 1981). It is interesting to see that, with the Ca/P/S-based CBS-400 implanted, the wall thickness of the new bone in the OVX_IP goat is comparable or even larger than that of the normal control goat.

The observed well-developed new bone network within the implantation site at 24 weeks post-implantation indicates that the implanted granules can probably help the skeleton to bridge the gap between the bone void and the native bone tissue in the course of healing and new bone formation processes. The porous morphology of the implant is believed to play a major role in the development of the new bone network. The largely interconnected pores built in individual granules and the gaps among granules both provide effective channels for vascular and new bone ingrowth (Klawitter and Hulbert, 1971) to form the desired trabecular network. From this point of view, the present CBS-400 implant seems to function like a scaffold that facilitates the attachment, spreading, division, and differentiation of cells. The designed high porosity (>70% v/v porosity) and appropriate pore size (100–200 μm) of the present CBS-400 granules provide interconnected pores to prevent the formation of blind alleys, which could lower the oxygen tension and influence osteoblast differentiation (Klawitter and Hulbert, 1971; D’Ippolito et al., 2006). The study of Hing et al. (2005) also suggested that a higher porosity in the implanted calcium phosphate might lead to a higher mineral apposition rate due to its increased permeability and angiogenesis.

In dealing with the frustrating osteoporosis-impaired bone healing process, the use of different anti-osteoporosis drugs or growth factors has been attempted to block the activity of osteoclast or induce osteoblastic differentiation which might promote new bone formation (Kyllönen et al., 2015). The use of biological supplementation, however, is not without risks/side effects. For example, the widely-used bisphosphonates may cause atypical femur fractures and the well-known osteonecrosis of the jaw (Khosla et al., 2007). The local addition of platelet-derived growth factor (PDGF) in fracture repair could promote growth of a variety of tumors (Yu et al., 2003). The side effects of BMP could include seroma formation, bone overgrowth, osteolysis and an increased risk of cancer (Hustedt and Blizzard, 2014).

It is worth noting that, in many articles investigating the healing effect of bone grafts or bone substitutes in osteoporotic bone, most of them compared the results to the osteoporotic control group, instead of comparing to a normal blank or sham control without osteoporosis treatment (Leung et al., 2006; Cao et al., 2012). Among the few authors who compared the graft-mediated new bone regeneration in osteoporotic animals with the healthy native bone, Blouin et al. (2006) compared the bone morphology of femoral diaphysis treated with injectable β-TCP/hydroxyapatite (HA)/hydroxypropyl methylcellulose composite to sham control in severely osteopenic rats. Their results indicate that, at 3 months post-implantation, the regenerated bone still show an apparently more porous structure than the healthy native bone with a 33% decrease in trabecular bone volume and a 34% decrease in trabecular bone number. Do Prado Ribeiro et al. (2012) also reported a weakened bone healing phenomenon in osteoporotic bone defects implanted with HA, compared to the non-osteoporotic control. In their study the quantity of HA-induced new bone in the osteoporotic bone was only 34% that of the sham group. Not only synthetic bone grafts, even the so-called “gold standard” autograft still shows a similar phenomenon when implanted in an osteoporotic bone. Li et al. (2010a) implanted autograft in the femur of both normal and osteoporotic rabbits and found that the new bone area in cancellous bone in the osteoporotic group was only 48% that of the sham group. Some recent studies indicate that the osteogenic differentiation of MSCs could be altered in osteoporotic bone (Xiao et al., 2007), which could change the fracture repair process in osteoporotic bone (Lu et al., 2005). Durão et al. (2014) investigated biomaterial-mediated healing of bone defects using a bovine bone (Bio-oss^®^) and observed an impaired differentiation and osteoblastic gene expression, which might at least partly explain its impaired bone healing behavior.

The above discussions clearly indicate that reestablishing normal bone repair in osteoporotic bone fracture is an ever-present challenge, as osteoporosis can delay the bone remodeling process, leaving more calluses or woven bones in the fracture region, and delay the new bone maturing to gain the desired mechanical properties (Walsh et al., 1997; Kubo et al., 1999; Meyer et al., 2001; Namkung-Matthai et al., 2001; Lill et al., 2003; McCann et al., 2008). With the proprietarily-designed material chemistry and structure of the present CBS-400 implant, the new bone architecture parameters in the OVX_IP goat become comparable to the normal control goat in about 6 months after implantation without signs of osteoporosis-related delay in the bone maturing process. The quick and nice recovery of the trabecular architecture parameters observed in the OVX_IP goat indicates that the present Ca/P/S-based bone substitute material, without the use of any anti-osteoporosis drug or growth factor, has a high potential to treat osteoporotic fractures.

## Conclusion

1. The present OVX procedure along with a low calcium diet and breeding away from light successfully induces osteoporosis in the female experimental goats.
2. The histological examination reveals a new trabecular bone network within the surgically-created defect of the OVX_IP goat, wherein numerous tiny implant residues are intimately embedded in the surrounding new bone throughout the newly-established lamellar bone structure. The newly-formed trabecular bone network in the OVX_IP goat appears much denser than the OVX goat and comparable to the healthy control goat. The transmitted polarized light microscopy reveals a more randomly-oriented, smaller-sized and apparently more active lamellar bone morphology in the OVX_IP goat as compared to the control and OVX goats. Few blood vessels are observed in the trabecular bone of the control and OVX goats. In the OVX_IP goat, however, numerous blood vessels and newly-formed trabecular packets with relatively thick walls surrounding implanted granules are observed. Compared to the control and OVX goats, the number of blood vessels is much larger, while the number of trabecular packets is smaller in the OVX_IP goat.
3. The results of histomorphometry and changes in trabecular architecture parameters show that, among all the experimental goats, the OVX_IP goat has the highest trabecular thickness and lowest trabecular bone packet prevalence, while the OVX goat has the lowest trabecular thickness and highest trabecular bone packet prevalence. The average trabecular bone tissue area ratio and trabecular number of the OVX goat are also significantly lower than the control and OVX_IP goats. On the other hand, the OVX goat has much larger average trabecular plate separation than the control and OVX_IP goats. The differences in trabecular plate separation, trabecular number and trabecular bone tissue area ratio between the OVX_IP goat and the control goat are not significant, indicating that the trabecular bone architecture of the CBS-400-implanted OVX_IP goat has substantially recovered to normal level in about 6 months after implantation without signs of osteoporosis-related delay in the bone maturing process. The quick recovery of the trabecular architecture parameters observed in the OVX_IP goat indicates that the present Ca/P/S-based bone substitute material, without the use of any anti-osteoporosis drug or growth factor, has a high potential to treat osteoporotic fractures.

## Data Availability Statement

The raw data supporting the conclusions of this article will be made available by the authors, without undue reservation, to any qualified researcher.

## Ethics Statement

The animal study was reviewed and approved by the Hengchun Branch, Livestock Research Institute, Council of Agriculture, Executive Yuan.

## Author Contributions

J-HC and C-PJ co-designed and supervised this study. B-CY conducted the histologic examination and histomorphometric analysis. S-ML operated the goat implantation. B-CY, C-PJ, and S-ML prepared the manuscript writing. All authors contributed to the article and approved the submitted version.

## Conflict of Interest

The authors declare that the research was conducted in the absence of any commercial or financial relationships that could be construed as a potential conflict of interest.
